# Digital spiral object identification using random light

**DOI:** 10.1038/lsa.2017.13

**Published:** 2017-07-28

**Authors:** Zhe Yang, Omar S Magaña-Loaiza, Mohammad Mirhosseini, Yiyu Zhou, Boshen Gao, Lu Gao, Seyed Mohammad Hashemi Rafsanjani, Gui-Lu Long, Robert W Boyd

**Affiliations:** 1 State Key Laboratory of Low-dimensional Quantum Physics and Department of Physics, Tsinghua University, Beijing 100084, China; 2 The Institute of Optics, University of Rochester, Rochester, New York 14627, USA; 3 School of Science, China University of Geosciences, Beijing 100083, China; 4 Tsinghua National Laboratory for Information Science and Technology, Beijing 100084, China; 5 Department of Physics, University of Ottawa, Ottawa ON K1N 6N5, Ontario, Canada

**Keywords:** object identification, orbital angular momentum, random light, remote sensing, second-order correlation

## Abstract

Photons that are entangled or correlated in orbital angular momentum have been extensively used for remote sensing, object identification and imaging. It has recently been demonstrated that intensity fluctuations give rise to the formation of correlations in the orbital angular momentum components and angular positions of random light. Here we demonstrate that the spatial signatures and phase information of an object with rotational symmetries can be identified using classical orbital angular momentum correlations in random light. The Fourier components imprinted in the digital spiral spectrum of the object, as measured through intensity correlations, unveil its spatial and phase information. Sharing similarities with conventional compressive sensing protocols that exploit sparsity to reduce the number of measurements required to reconstruct a signal, our technique allows sensing of an object with fewer measurements than other schemes that use pixel-by-pixel imaging. One remarkable advantage of our technique is that it does not require the preparation of fragile quantum states of light and operates at both low- and high-light levels. In addition, our technique is robust against environmental noise, a fundamental feature of any realistic scheme for remote sensing.

## Introduction

The orbital angular momentum (OAM) of light has attracted considerable attention in recent years. As identified by Allen *et al.*^1^ in 1992, a beam of light with an azimuthal phase dependence of the form 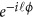 carries OAM, where 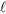 is the mode index, which specifies the amount of OAM, and *Φ* is the azimuthal angle. This interesting property of light has been explored in different contexts. For example, fundamental tests of high-dimensional entangled systems have been performed through the OAM basis^2^, the infinite OAM bases have been used to implement paradoxes in quantum mechanics^3^ and relativistic effects have been explored in the azimuthal degree of freedom^4, 5^. In the applied context, the OAM of light has been used to encode information^6, 7, 8, 9, 10^, manipulate microscopic particles^11, 12, 13, 14^, perform optical metrology^15, 16^, and perform remote sensing and imaging^4, 17, 18, 19, 20, 21, 22, 23, 24, 25^.

It has been suggested that the discrete OAM spectrum (or spiral spectrum) can be used for imaging, a technique known as digital spiral imaging^17^. In addition, quantum OAM correlations^26^ have been used to enhance the image contrast of phase objects^18^. Furthermore, object identification has been performed by means of quantum-correlated OAM states^19, 20^. Similarly, quantum correlations have been incorporated into digital spiral imaging to retrieve information of phase objects^21^. Moreover, field correlations in vectorial beams have been utilized for kinematic sensing^22^.

It has been recently demonstrated that random fluctuations of light give rise to the formation of intensity correlations in the OAM components and angular positions of pseudothermal light^27^. It has also been shown that these classical correlations are manifested through interference structures that resemble those observed with entangled photons. These results suggest that OAM intensity correlations in random optical fields, such as those found in natural light, could be used to develop optical technologies with the similar functionality as those that employ entangled photons.

In this work, we exploit the OAM correlations of random light to demonstrate object identification; in this approach, the object is identified through its discrete OAM spectrum (or spiral spectrum). We also demonstrate that we can use the same types of correlations to retrieve the phase information of an object. Despite the fact that intensity correlations in the OAM degree of freedom are not perfect, as for the case of entangled photons, it is possible to perform object identification at any light levels, an important advantage over the quantum protocols that employ fragile entangled states of light.

## Materials and methods

### Theoretical analysis

The OAM spectrum of a random light field *E*(*r*,*Φ*) can be experimentally measured by projecting it onto a series of OAM modes 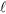. The amplitude for this projection is given by


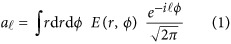


The angular coherence properties of a field of light are described by the first-order correlation function 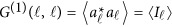, where the symbol 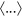 indicates the ensemble average. Similarly, the second-order correlation function that describes intensity correlations in the OAM domain is defined as 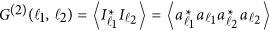. For a thermal beam of light, 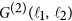 is given by^27, 28^


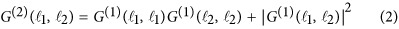


The first term in Equation (2) represents a product of intensities between two OAM modes. This first term is constant and represents a background that causes the intensity correlations not to be perfect, whereas the second term, 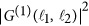, is typically approximated by a discrete *δ* function that describes point-to-point OAM correlations.

In our scheme for object identification, one of the two beams illuminates the object described by the transmission function *A*(*r*,*Φ*). In this case, the second term of Equation (2), which is defined as 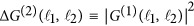, can be expressed (see the Supplementary Information) as


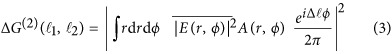


where 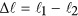, and the overbar means ensemble average. Interestingly, the object *A*(*r*,*Φ*) encodes its Fourier components into the second-order correlation function. This signature is used to recover its spatial or phase information. When the object is not present, *A*(*r*,*Φ*)=1, and this term takes the form of a discrete *δ* function.

### Experimental setup

**Figure 1:**
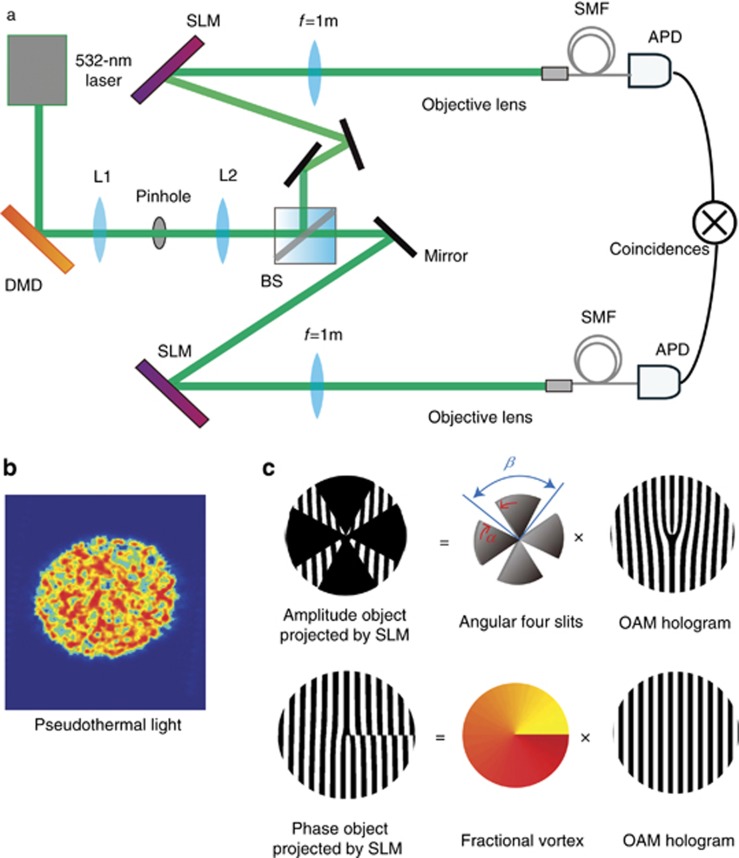
Experimental setup for digital spiral object identification with random light. (**a**) Experimental setup. A DMD is illuminated by a 532-nm laser beam. The first diffraction order of the structured beam is isolated by a 4*f*-optical system comprised of two lenses and a spatial filter in the focal plane (figure not to scale). A series of random patterns are displayed on the DMD at a frequency of 1.4 kHz to produce a random field of light. The generated beam is divided by a beam splitter to produce a test beam that interacts with the object and a reference beam. An SLM in each arm is used to measure the OAM components in the random beam of light. (**b**) Image of the spatial intensity distribution of the random beam of light. (**c**) The amplitude or phase object is encoded into the SLM in the test arm. SMF, single mode fiber.

Figure 1a shows the experimental setup we use for digital spiral object identification. A 532-nm diode laser illuminates a digital micro-mirror device (DMD), which is used to generate a random field of light^29, 30^. A 4*f*-optical system consisting of two lenses and a spatial filter is employed to isolate the first order of the beam diffracted by the DMD. The intensity distribution of the generated beam is shown in Figure 1b.

The random light field is divided into ‘test’ and ‘reference’ arms after passing through a beam splitter. The light beam in the test arm interacts with an amplitude or phase object, which is displayed onto a spatial light modulator (SLM), as shown in Figure 1c. Each light beam is then projected onto a forked hologram to measure an OAM component of the random field of light^19, 20, 21, 31^. The first diffraction order of the structured beam is filtered by an aperture and then is coupled into a single mode fiber and detected by an avalanche photodiode (APD). Two APDs and a coincidence count module are utilized to measure OAM correlations between the two arms. The total accumulation time of each measurement is set to 5 s in our experiment.

## Results and discussion

### Amplitude object identification

**Figure 2:**
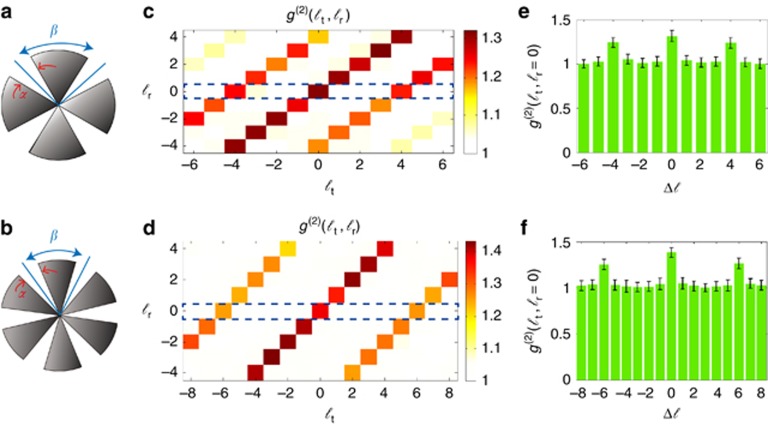
Digital spiral identification for amplitude objects with four- and sixfold rotational symmetries. (**a**) An object with fourfold rotational symmetry with *α*=*π*/6 and *β*=*π*/4. (**b**) A similar object with *α*=*π*/8 and *β*=*π*/3. (**c**, **d**) The corresponding second-order correlation matrices. (**e**, **f**) The rows denoted by the dotted boxes in **c** and **d**, respectively.

As shown in Figure 2a and 2b, we use objects with four- and sixfold rotational symmetries. Each object is encoded onto the SLM located in the test arm.

A series of OAM projections is performed in each arm to construct a two-dimensional matrix with the normalized second-order correlation function (Figure 2c and 2d). The OAM number in the test and reference arms are denoted by 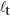 and 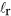, respectively. The normalized second-order OAM correlation function is calculated by 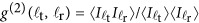, where 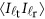 is proportional to the coincidence count rate. Each element in the matrix is obtained by averaging over 50 realizations of the experiment, and the error bars are obtained by calculating the standard deviation.

As shown in Figure 2c and 2d, an amplitude object with *N*-fold rotational symmetry imprints its Fourier components into the second-order OAM correlation matrix. The correlation signal is high along the diagonal elements of the matrix, where 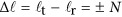 due to the symmetry of the amplitude object. In our case, these signatures can be observed when 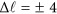, for the object with fourfold rotational symmetry, and when 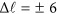, for the object with sixfold rotational symmetry. Consequently, it is evident that one can use the OAM correlation matrix to identify the two objects. Furthermore, note that this technique requires a small number of measurements compared to traditional imaging schemes that rely on pixel-by-pixel raster scanning.

In Figure 2e and 2f, we plot the transverse sections, defined by 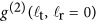, for the correlation matrices in Figure 2c and 2d, respectively. For simple and symmetric objects, a single line in the correlation matrix can provide adequate information about the object. However, the measurement of the total OAM correlation matrix is required for complicated objects that lack rotational symmetry^19, 32^.

### Phase object identification

**Figure 3:**
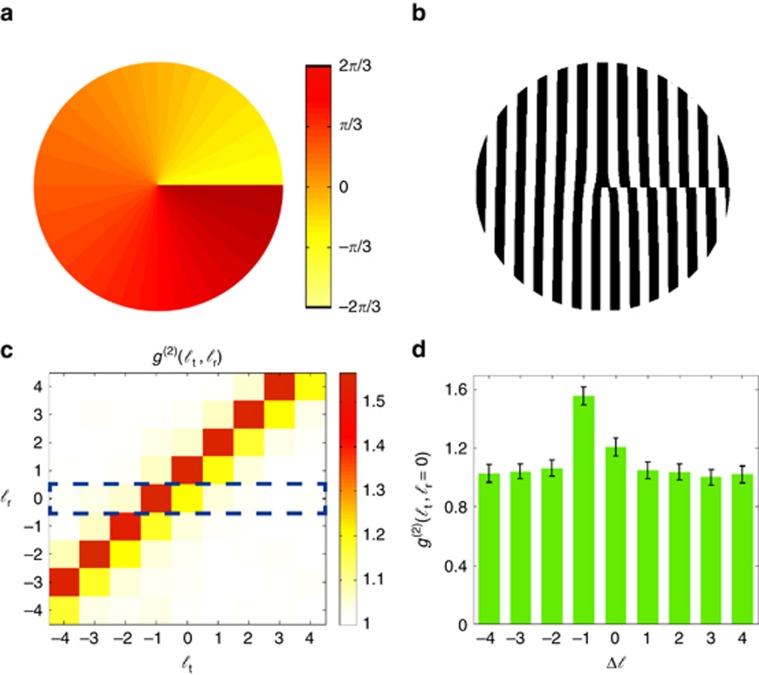
Digital spiral identification for a phase object. (**a**) The phase object consisting on a non-integer vortex with an OAM number of *M=*−2/3. (**b**) The corresponding forked hologram that we encode onto the SLM located in the test beam. (**c**) Experimental results for the second-order OAM correlation matrix. (**d**) A plot of the row denoted by the dotted box in **c**.

We showed above that our technique is capable of identifying amplitude objects with rotational symmetry. Next, we demonstrate that our technique can also be used to identify phase objects. As a specific example, we use phase objects consisting of non-integer vortices described as *e*^−*iMΦ*^, where *M* indicates a non-integer winding number^33, 34, 35, 36, 37^. The phase profile of a vortex with *M*=−2/3 is shown in Figure 3a; the azimuthal phase for a non-integer vortex of this form ranges from −2*π*/3 to 2*π*/3. The forked hologram that we encode onto the SLM is shown in Figure 3b. The two-dimensional normalized second-order OAM correlation matrix is shown in Figure 3c, and its middle row is plotted in Figure 3d. In this case, the presence of the phase object induces a broader spectrum in the correlation matrix.

**Figure 4:**
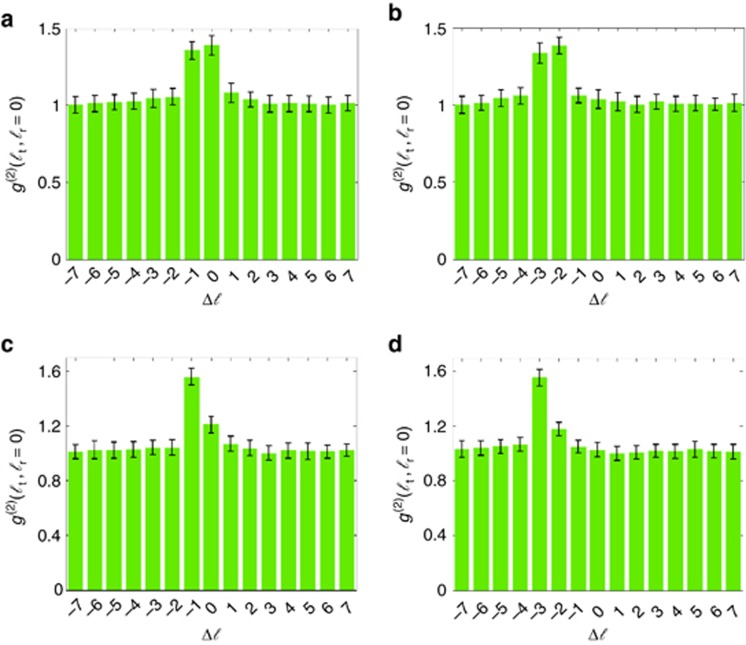
Digital spiral identification for phase objects with different non-integer winding numbers: (**a**) *M*=−1/2, (**b**) *M*=−5/2, (**c**) *M*=−2/3 and (**d**) *M*=−8/3.

As shown in Figure 4, we also test the performance of our technique with different phase objects characterized by the non-integer winding numbers *M*=−1/2, *M*=−5/2, *M*=−2/3 and *M*=−8/3. The performance of our technique can be characterized through the Floor function. This simple function is used to denote the largest previous integer of *M* and can be defined as 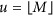, and *v* is the non-integer part given by *v=M−u*. The theoretical and experimental results show that the central peak of the correlation signal is determined by *u* and the profile is determined by *v*. A simple comparison between Figure 4a and 4b shows that the two figures have the same profile, but the central peak is located at different positions. This difference is because the parameter *v* is equal to 1/2 for both cases, whereas the parameter *u* is different; this parameter is equal to −1 and −3 for Figure 4a and 4b, respectively. We can compare the results shown in Figure 4c and 4d; in this case, the parameter *v* is equal to 1/3, whereas the parameter *u* is equal to −1 and −3 for Figure 4c and 4d, respectively. In this case, the two figures have the same profile, but the peak is located at different positions.

In our experiment, we used phase objects consisting of non-integer vortices. However, this technique can be applied to the identification of other phase objects, such as those discussed in Refs 38, 39, 40. These schemes require coherent sources of light or entangled photons.

The use of random light and intensity correlations in our technique shares similarities with other techniques, such as thermal ghost imaging^41^. However, our technique extracts the fingerprints that characterize objects with rotational symmetries, leading to a reduction in the number of measurements required in conventional techniques for imaging. Another interesting aspect of our technique is that the second-order interference effects are less sensitive to the coherence properties of the source. In fact, this is one of the advantages of the Hanbury Brown and Twiss interferometer compared to the Michelson interferometer^42^. In addition, it has been demonstrated that imaging schemes based on second-order correlations are robust against turbulance^43^.

## Conclusions

We experimentally demonstrated digital spiral object identification for an amplitude and a phase object using second-order OAM correlations with random light. In our technique, the object imprints its Fourier components onto the digital spiral spectrum; by measuring intensity correlations in the OAM degree of freedom, we can retrieve spatial and phase information for different masks. Compared to conventional pixel-by-pixel imaging, this technique only requires a small fraction of the number of measurements to identify an object; this peculiarity makes our technique sparse sensitive, similar to other techniques that rely on compressive sensing. In addition, our technique does not rely on fragile quantum states of light and can operate at low- and high-light levels. Finally, our technique is robust against environmental noise and has potential applications in remote sensing and imaging.

## Author contributions

OSM-L conceived the idea. The experiment was designed by ZY, OSM-L, MM, GL and RWB. The theoretical description of our work was developed by ZY, BG and SMHR. The experiment was performed by ZY, YZ, LG, OSM-L and MM. The data were analyzed by ZY, with help from OSM-L. The project was supervised by GL and RWB. All authors contributed to the discussion of the results and to the writing of the manuscript.
