# Supplementary material for: Digital spiral object identification using random light
Source: Light Sci Appl. 2017 Jul 28;6(7):e17013–. doi: 10.1038/lsa.2017.13 (PMC6062229; doi:10.1038/lsa.2017.13)
Supplement: Supplementary Material [file lsa201713x1.docx]

Supplementary Information for

Digital spiral object identification using random light

Zhe Yang^1,2^, Omar S Magaña-Loaiza^2,†^, Mohammad Mirhosseini^2^, Yiyu Zhou^2^, Boshen Gao^2^, Lu Gao^2,3^, Seyed Mohammad Hashemi Rafsanjani^2^, Gui-Lu Long^1,4,†^ and Robert W Boyd^2,5^ 0F[[1]](#footnote-1)↵

1. **Second-order OAM correlations between two beams**

We describe a thermal light field in the polar coordinate by . Experimentally, the thermal light field can be projected onto a series of OAM modes  to measure the corresponding OAM spectrum. The field amplitude of this measurement is given by

(1)

The first-order OAM correlation function describes the angular coherence properties of the light field.

If $l_{1}=l_{2}$, $G^{(1)}(l,l)$ represents the intensity of the light field projected onto the OAM mode

(2)

The ensemble average spectral intensity can be described as

(3)

If the transverse coherence length of the random light field is small enough, one get the relation

(4)

The ensemble average spectral intensity can be expressed as

(5)

which is independent on winding number  of OAM.

If an object is placed in the light beam, the light field takes the form of where is the transmission function of the object. The thermal light field passing through an arbitrary amplitude and phase object can be expressed in terms of angular harmonics that . The coefficients can be calculated by. Therefore, the average spectral intensity of this beam reads as

(6)

Similar to Eq. (5), this term is also independent on , consequently, one can not obtain any information of the object from the digital spiral spectrum of a single thermal beam of light.

The second-order OAM correlation between two beams is defined as , and this takes the form of

(7)

Therefore, the ensemble average of the second-order OAM correlation reads as

(8)

For a thermal light field, the second-order correlation can be expressed in terms of first-order correlation functions

(9)

Combining Eq. (8) and Eq. (9), one can obtain the second-order OAM correlation

(10)

where the first term is the background which describes the ensemble average spectral intensities of two beams. The second term, which is given by is the signal taking the form of

(11)

where .

1. **Identification for amplitude object**

Now we discuss the identification for an amplitude object with *N*-fold rotational symmetry. The transmission function of the object with *N* periodic slits takes the form of

(12)

where *n*=*0,1,2,...N-1*, $\alpha$ is the width of one angular slit and $\beta=2\pi/N$ is the spacing between the centers of two adjacent angular slits.

Substituting Eq. (12) into Eq. (11), the signal term is given by

$G^{(2)}(l_{1},l_{2})\sim\left\langle I_{l_{1}}\right\rangle\left\langle I_{l_{2}}\right\rangle\left| \sum_{n=0}^{N-1} e^{i\frac{n\Delta l\beta}{2}}\right|^{2}\frac{\alpha^{2}}{(2\pi)^{2}}\sin c^{2}(\frac{\Delta l\alpha}{2}).$ (13)

The amplitude object with *N*-fold rotational symmetry imprints its particular signature to the OAM spectrum, from which we can be obtained the information of the object.

1. **Identification for phase object**

In the following analysis, the non-integer vortex (fractional vortex) is treated as the phase object. The non-integer vortex can be described by, where *M* is not an integer and the corresponding second-order OAM correlation signal term is given by

(14)

If the Floor function is used to denote the largest previous integer of *M*, i.e., $u=\left\lfloor M\right\rfloor$, and *v=M-u* is the proper fractional part, one can arrive at

. (15)

This equation shows that the peak position of this term is determined by *u* and the spread distribution in the OAM spectrum is given by *v*.

1. 1 State Key Laboratory of Low-dimensional Quantum Physics and Department of Physics, Tsinghua University, Beijing 100084, China

   2 The Institute of Optics, University of Rochester, Rochester, New York 14627, USA

   3 School of Science, China University of Geosciences, Beijing 100083, China

   4 Tsinghua National Laboratory for Information Science and Technology, Beijing 100084, China

   5 Department of Physics, University of Ottawa, Ottawa, ON K1N 6N5, Canada

   †Correspondence: Omar S Magaña-Loaiza, E-mail: omar.maganaloaiza@rochester.edu

   †Correspondence: Gui-Lu Long, E-mail: gllong@mail.tsinghua.edu.cn [↑](#footnote-ref-1)
